# Accelerated craniofacial bone regeneration through dense collagen gel scaffolds seeded with dental pulp stem cells

**DOI:** 10.1038/srep38814

**Published:** 2016-12-09

**Authors:** Frédéric Chamieh, Anne-Margaux Collignon, Benjamin R. Coyac, Julie Lesieur, Sandy Ribes, Jérémy Sadoine, Annie Llorens, Antonino Nicoletti, Didier Letourneur, Marie-Laure Colombier, Showan N. Nazhat, Philippe Bouchard, Catherine Chaussain, Gael Y. Rochefort

**Affiliations:** 1 EA 2496 Orofacial pathologies, imaging and biotherapies, Dental School Faculty, University Paris Descartes, and Life imaging Platform (PIV), Montrouge, France; 2 Department of Periodontology, Service of Odontology, Rothschild Hospital, AP-HP, University Denis Diderot, U.F.R. of Odontology, Paris, France; 3 Department of Odontology, University Hospitals, AP-HP, Paris, France; 4 INSERM U1148, Laboratory of Vascular Translational Science, University Paris Diderot, University Paris 13, Sorbonne Paris Cité, X Bichat Hospital, and Département Hospitalo-Universitaire (DHU) FIRE, F-75018 Paris, France; 5 Department of Mining and Materials Engineering, McGill University, Montreal, Quebec, Canada

## Abstract

Therapies using mesenchymal stem cell (MSC) seeded scaffolds may be applicable to various fields of regenerative medicine, including craniomaxillofacial surgery. Plastic compression of collagen scaffolds seeded with MSC has been shown to enhance the osteogenic differentiation of MSC as it increases the collagen fibrillary density. The aim of the present study was to evaluate the osteogenic effects of dense collagen gel scaffolds seeded with mesenchymal dental pulp stem cells (DPSC) on bone regeneration in a rat critical-size calvarial defect model. Two symmetrical full-thickness defects were created (5 mm diameter) and filled with either a rat DPSC-containing dense collagen gel scaffold (n = 15), or an acellular scaffold (n = 15). Animals were imaged *in vivo* by microcomputer tomography (Micro-CT) once a week during 5 weeks, whereas some animals were sacrificed each week for histology and histomorphometry analysis. Bone mineral density and bone micro-architectural parameters were significantly increased when DPSC-seeded scaffolds were used. Histological and histomorphometrical data also revealed significant increases in fibrous connective and mineralized tissue volume when DPSC-seeded scaffolds were used, associated with expression of type I collagen, osteoblast-associated alkaline phosphatase and osteoclastic-related tartrate-resistant acid phosphatase. Results demonstrate the potential of DPSC-loaded-dense collagen gel scaffolds to benefit of bone healing process.

Tissue engineering approaches offer novel treatment modalities in numerous medical disciplines^1^, including bone augmentation procedures in oral surgery for dental implant placement and periodontal reconstructions. While autologous bone grafting is the current “gold standard” procedure in large defects^2^, it exhibits some limitations such as availability of sufficient graft volume, donor site morbidity or unpredictable bone resorption^3^,^4^. On the other hand, allografts or xenografts have been shown to be associated with immunoreactions, and a risk of transmission of either bacteria or viruses^5^,^6^. Although synthetic grafts can be easily absorbed, they do not exhibit osteoinductive properties. To overcome the drawbacks associated with grafting procedures, current bone tissue-engineering strategies are employing different combinations of osteoconductive substitutes, growth factors and stem/progenitor cells^7^. These biomaterial templates aim to not only provide an adequate volume for the prevention of soft tissue collapse into the intrabony defect, but also stimulate seeded cell migration, proliferation and differentiation through biological and mechanical cues.

A large variety of biomaterials has been used as carriers in bone tissue engineering approaches, including the widely used three dimensional (3D) collagen-based biomimetic hydrogel scaffolds^8^,^9^. These hydrogel scaffolds are biocompatible, biodegradable with low antigenicity, which provide a favorable environment to support osteoblast attachment, proliferation, and differentiation^10^,^11^. Although collagen scaffolds are *per se* highly hydrated (with more than 95% w/v fluid) with weak mechanical properties for tissue replacement applications^12^, the “simple” plastic compression of the material rapidly increases the relative collagen fibrillar density (to more than 10% in weight) by removing the excess of fluid^13^. The “plastic compression” approach thus yields a type I collagen matrix with a fibrillar density similar to that of native bone matrix^14^,^15^,^16^. This process enables the rapid, controllable and reproducible production of dense collagen gel scaffolds with highly defined meso-structure and increased biomechanical properties, similar to that of the osteoid^10^,^17^,^18^. Furthermore, cell seeding constitutes part of the processing route, and the scaffolds provide the 3D structure for their growth and differentiation without compromising their viability^13^,^19^.

Mesenchymal stem cells (MSCs) are common candidates for scaffold-based tissue engineering^20^. Dental pulp stem cells (DPSCs) are neural crests derived cells^21^,^22^, which exhibit MSC characteristics and have the ability to differentiate into odontoblasts, adipocytes, osteoblasts, chondrocytes and myocytes^23^,^24^,^25^,^26^. Dental stem cells can be harvested from several dental sources, including DPSCs, stem cells from human exfoliated deciduous teeth (SHED), stem cells from the apical papilla (SCAP), periodontal ligament stem cells (PDLSC), and dental follicle precursor cells (DFSC)^27^. In particular, DPSCs are a potential alternative source for bone regeneration/healing and tissue engineering attributable to their high proliferation rates, their extended differentiation potential, and paracrine properties^28^,^29^,^30^. In addition, similarly to MSCs that have been shown to exhibit immunomodulatory properties in syngeneic, allogeneic and xenogeneic applications^31^, human DPSC transplanted into large rat calvarial defects have been demonstrated to differentiate into osteogenic cells without any graft rejection^32^. Thus, we hypothesized that dense collagen scaffold, which constitutes a physiological osteogenic extracellular matrix due to its elevated fibrillar density, combined with mesenchymal stem cells derived from the dental pulp, would enhance bone regeneration. Therefore, the aim of this study was to evaluate the osteogenic effects of dense collagen gel scaffolds seeded with rat DPSC (rDPSC) implanted in a rat critical-sized calvarial defect model. The bone repair process was dynamically monitored *in vivo*, using micro-computed tomography and analyzed by histomorphometry.

## Material and Methods

### Ethical approval and animal management

All experiments in this study were designed according to ARRIVE guidelines, and performed with a protocol approved by the Animal Care Committee of the University Paris Descartes (No. P2.JLS.174.10). Animals were maintained according to the guidelines for ethical conduct developed by the European Communities Council Directive (animal breeding agreement C92-049-01). All efforts were made to minimize their pain or discomfort.

WISTAR rats were purchased from Janvier Labs (Le Genest Saint Isle, France). They were housed at stable conditions (22 ± 2 °C) with a 12 h dark/light cycle, and with ad libitum access to water and food in the animal facility of the Department of Orofacial pathologies, imagery and biotherapies of Descartes University, Montrouge, France.

### Isolation and culture of rat Dental Pulp Stem Cells

Multi-colony-derived rat dental pulp stem cells (rDPSCs) were obtained using a protocol adapted from Gronthos *et al*. from the molars of 4-day Wistar rats^33^. Under sterile conditions, rat molars were extracted and incubated at 4 °C for 45 min in phosphate-buffered saline (PBS) containing 100 U/mL penicillin/streptomycin (Gibco) and 250 μg/mL fungizone (Gibco), then in PBS containing 3 mg/mL type I collagenase (Worthington Biochem) and 2 U/mL dispase I (Roche) in a shaking incubator (at 37 °C) for 1 h. The isolated cells were then plated on 0.1% gelatin-coated dishes in the Minimum Essential Media-alpha (Gibco) supplemented with 20% v/v fetal bovine serum (FBS) (Gibco) and 100 U/mL Penicillin/streptomycin (Gibco), 1 ng/ml FGF-2 (Peprotech), and maintained at 37 °C under 5% CO_2_ atmosphere. The medium was changed after 2 days, and then twice a week. Upon reaching 70–80% confluence, cells were plated at 10^4^ cells per cm^2^. The medium was changed after 2 days, and then twice a week. The required cell number for the *in vivo* experiments was reached after 2–3 passages.

### Rat Dental Pulp Stem Cell Phenotype by Flow Cytometry

The expression of CD31 (AF488, #MCA1334A488, BioRad, Oxford, UK), CD45 (PE, #MCA43PE, BioRad), CD73 (eF450, #48-0731-82, eBioscience Inc., San Diego, CA) and CD90 (PE-Cy5, #ab95809, Abcam, Cambridge, UK) was analyzed by polychromatic flow cytometry (LSRII; Becton Dickinson, Franklin Lakes, NJ) with fluorochrome-conjugated monoclonal antibodies. Cells at passage 2 were detached by 4% lidocaine (Sigma, St Louis, MO). BD CompBeads particles (BD Biosciences) were used to calculate the compensation of fluorescence spillover. Only events from alive cells in both morphogate and singulet gates were analyzed.

### Scaffold preparation

Plastically compressed collagen gels were used as 3D scaffolds and prepared as previously described^10^,^13^,^34^. The polymerizing type I collagen gel prepared solution (rat-tail tendon in 0.1% v/v acetic acid solution, neutralized by NaOH and obtained as previously described^35^, 1.6 mg/ml final) acellular or with rDPSCs at a seeding density of 2 × 10^6^ cell/mL was ice-cold mixed and platted into a 4-well plate. After polymerization (30 min at 37 °C), highly hydrated hydrogels, with a fibrillar collagen density of less that 0.5% w/v, were placed on a stack of blotting paper, nylon, and stainless steel meshes. Dense collagen gel scaffolds, with fibrillar collagen density of more than 10% w/v^13^, were produced by the application of an unconfined compressive stress of 1 kPa for 5 min to remove excess casting fluid. The compressed scaffolds were circularly cut (5 mm diameter) and kept up to 24 h at 37 °C under 5% CO_2_ in serum-free medium before implantation.

### Surgical implantation, experimental procedure and sampling

Wistar male rats (12 week-old, ~300 g) were anesthetized (100 mg/kg b.w. of ketamine and 10 mg/kg b.w. of xylazine hydrochloride, both from Centravet Alfort, Maisons-Alfort, France). In each specimen, scalp skin was incised, and the periosteum was eliminated to visualize the skull. A 5-mm-diameter calvarial critical-sized defect was created on each side of the parietal bone using a dental bur attached to a slow-speed hand piece operating at 1500 rpm, under irrigation with sterile saline solution^36^. Special care was taken for the sagittal suture preservation, and minimal invasion of the dura mater. After gently removing the circular bone plug, a rDPSC-seeded dense gel scaffold (n = 15) was placed on one side of the rat skull and an acellular dense scaffold (n = 15) was placed on the opposite side, each rat being its own control. Defects created in the skulls of two additional rats were left empty as negative controls for the critical-size defect. Wound closure was achieved by a two layer suturing (periosteum, skin) using absorbable sutures (Vicryl Rapid 5.0 and 4.0 respectively, Ethicon, Johnson & Johnson). Immediate post-operative care included analgesia with buprenorphine (0.02 mg/kg b.w.). After surgery, the animals were housed individually under constant conditions. No lethality was detected during the surgery or the post-operative period. Wound healing progressed without any sign of infection, material exposure or other complication. Body weights were examined regularly to ensure proper feeding before and after surgery.

At days 7, 14, 21, 28 and 35 post surgery, animals were imaged using a micro-CT as described below. Three animals were euthanized at each time point, the calvarium was excised and separated into hemicalvarium. Samples were fixed in 70% v/v ethanol (24 hours at 4 °C), dehydrated in graded ethanol solutions, and embedded at −20 °C in methyl methacrylate resin (Merck) without decalcification^37^. Resin embedded hemicalvarium bone samples were cut (5 μm thick) using a Jung Polycut E microtome (Leica) with hard tissue blades (Leica). After immersion in a drop of 50% v/v ethanol, sections were stretched to a fold-free state on polysine glass slides (Menzel-Gläser), covered with a polyethylene sheet, and tightly pressed on the glass slides, followed by overnight drying at RT. Deplastification was carried out in 2-methoxyethyl acetate (Carlo Erba) three times for 20 minutes. Rehydration of the sections was performed in graded ethanol solutions for subsequent procedures.

### Micro-X-ray computed tomography (Micro-CT) examination of samples

For bone regeneration exploration, rats were anesthetized (isoflurane, induction at 3–4% under airflow of 0.8–1.5 L/min; 1.5–2% under 400–800 ml/min thereafter). They were imaged using an X-ray micro-CT device (Quantum FX Caliper, Life Sciences, Perkin Elmer, Waltham, MA) hosted by the PIV Platform, EA2496, Montrouge, France. The X-ray source was set at 90 kV and 160 μA. Tridimensional images were acquired with an isotropic voxel size of 20 μm. An internal density phantom, calibrated in mg of hydroxyapatite, was used to scale bone density. Full 3D high-resolution raw data are obtained by rotating both the X-ray source and the flat panel detector 360° around the sample (scanning time: 3 min). Tridimensional rendering were subsequently extracted from Dicom data frames using the open-source OsiriX imaging software (v5.7.1, distributed under LGPL license, Dr A. Rosset, Geneva, Switzerland). Quantification of the regenerated bone inside each defect was done using CTscan Analyzer software (Skyscan, release 1.13.5.1, Kontich, Belgium). A global volume of interest (VOI) was drowning by interpolating 2D region of interests on consecutive sections to isolate the defect area. The obtained interpolated VOI comprised only the remodeled bone defect area. A global thresholding was determined interactively for bone selection and to eliminate background noise. The following parameters were analyzed: bone density (in mg of hydroxyapatite), bone volume fraction BV/TV (%), trabecular thickness Tb.Th (mm), trabecular number Tb.N (1/mm), trabecular separation Tb.Sp (mm) and trabecular pattern factor Tb.Pf (1/mm)^38^.

### Histology examination of samples and histomorphometry

Deplastified hemicalvarium bone sample sections, (5 μm thick) were sequentially cleared in water and stained with toluidine blue (pH 3.8), Von Kossa staining, Sirius red, or processed for alkaline phosphatase (ALP) enzymohistochemistry and for tartrate-resistant acid phosphatase (TRAP) revelation. Toluidine blue staining was used to visualize connective bone matrix. Von Kossa staining was used to visualize mineralized bone. Sirius red staining was used to visualize birefringent Type I collagen fibers in the bone marrow area. TRAP was detected by using hexazotized pararosanilin (Sigma) and naphtol ASTR phosphate (Sigma, St Louis, MO) to reveal osteoclasts; non-osteoclastic acid phosphatase was inhibited by adding 100 mMol/L L(+)-tartric acid (Sigma, St Louis, MO) to the substrate solution. Image acquisition was performed using a DMLB Leica microscope, equipped with imaging camera DFC425 Leica connected to the Leica application (LAS version 4.4) and image analysis was performed using ImageJ.

### *In situ* hybridization analysis

RNA extractions were performed using RNeasy Plus mini kit (Qiagen, Venlo, Netherlands), and cDNA were synthesized using the Verso cDNA synthesis kit (ThermoFicher, Waltham, MA). RNA probes were designed using SP6 and T7-tailed oligonucleotides for Col1a1 (forward primer 5′ATTTAGGTGACACTATAGGAACAAGGTGACAGAGGCAT 3′ and reverse primer 5′ TAATACGACTCACTATAGGGTTGGTTAGGGTCGATCCAGT 3′), for a total length amplified between primers of 540 bp. Sense and antisens RNA probes were synthesized and labeled with Digoxigenin-UTP (Roche, Inc.) using the Riboprobe^®^ Combination System –SP6/T7 RNA Polymerase kit (Promega) following the manufacturer’s instructions. The mixture contained the following nucleotides: 0.1 mM of rATP, 0.1 mM of rCTP, 0.1 mM of rGTP, 0.05 mM of rUTP, 0.05 mM of digoxigenin-11-dUTP (Roche), and 800 ng of rat cDNA as templates. Probes were purified using the kit Illustrated ProbeQuant G-50 Micro Columns (GE Healthcare).

Deplastified and PBS 1X rehydrated hemi-calvarium bone sample sections (5 μm thick) were permeabilized with 10 μg/ml proteinase K (Sigma) in PBS at 37 °C for 20 minutes, and post-fixed in 4% neutral-buffered formalin for 30 minutes at room temperature. After washing in PBS for 5 minutes, followed by washes SCC 2X, hybridization was performed in a humid chamber at 65 °C overnight in a mixture containing the labeled RNA probe at 1/100 in 1 mg/ml Yeast ARNt, 50% formamide, 10% dextran sulfate, SCC 5X and Denhardt’s 1X. After hybridization, excess probe was eliminated by washing the sections for 30 minutes in buffer with 50% formamide in SCC 1X and 0.1% Tween 20 at 65 °C for 30 min, then twice for 1 hour. Then, tissue sections were equilibrated in a bath of MABT (100 mM maleic acid, 0.5 M NaCl, 0.1% Tween 20) at room temperature for 30 minutes. After 2 hours in blocking solution (20% inactivated goat serum, 2% Blocking Reagent [Roche] in MABT), the presence of Col1a1 in tissue sections was researched by immunoreactivity with an anti-digoxigenin-AP, Fab fragments (Roche Diagnostic) at 1/1000 in blocking solution, overnight at room temperature. After rinsing in MABT five times for 30 minutes, the presence of Col1a1 in tissue sections was revealed using NBT (0.45 μl/ml, Roche) and BCIP (3.5 μl/ml, Roche). The tissue sections were then rinsed and covered with a coverslip mounted Gel Mounting Medium (Dako Cytomation). Image acquisition was performed using a DMLB Leica microscope, equipped with imaging camera DFC425 Leica connected to the Leica application (LAS version 4.4).

### Statistical analysis

Results in each group were expressed as the mean ± standard error of the mean (S.E.M.). Variables were compared using one-way analysis of variance (ANOVA) followed with two-by-two comparisons performed with paired T-test of Student when they passed the Fisher F equal variance and Shapiro-Wilk normality tests. Otherwise, they were performed using Kruskal-Wallis ANOVA on the ranks followed with two-by-two comparisons performed with the U test of Mann and Whitney. Statistical significance was set at p < 0.05.

## Results

### rDPSCs displayed a mesenchymal stromal cell phenotype

**Figure 1:**
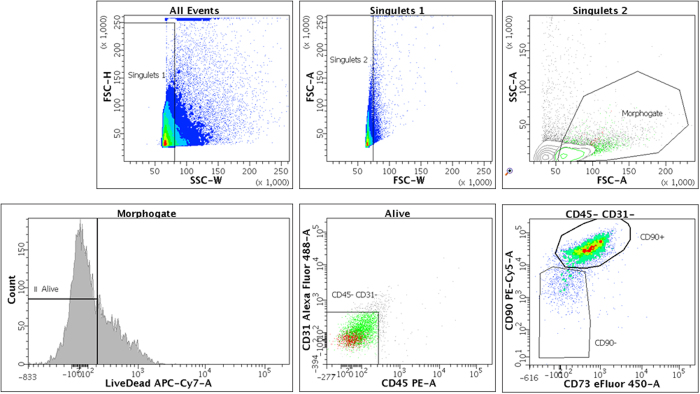
rDPSCs phenotype by flow cytometry. The expression of a series of cell surface markers associated with the mesenchymal stem cell (MSC) phenotype was investigated using flow cytometry. Passage 2 rDPSCs, represented by events from alive cells in both morphogate and singulet gates, were negative for CD31 and CD45, and positive for CD73 and CD90, as expected for mesenchymal stromal cells.

The expression of a series of cell surface markers associated with the mesenchymal stem cell (MSC) phenotype was investigated using flow cytometry. Most of passage 2 rDPSCs, represented by events from alive cells in both morphogate and singulet gates, were negative for CD31 and CD45, and >75% were positive for CD73 and CD90, as expected for mesenchymal stromal cells (Fig. 1)^39^,^40^.

### Dense collagen gel scaffolds seeded with rDPSCs improved bone healing process under Micro-X-ray computed tomography

**Figure 2:**
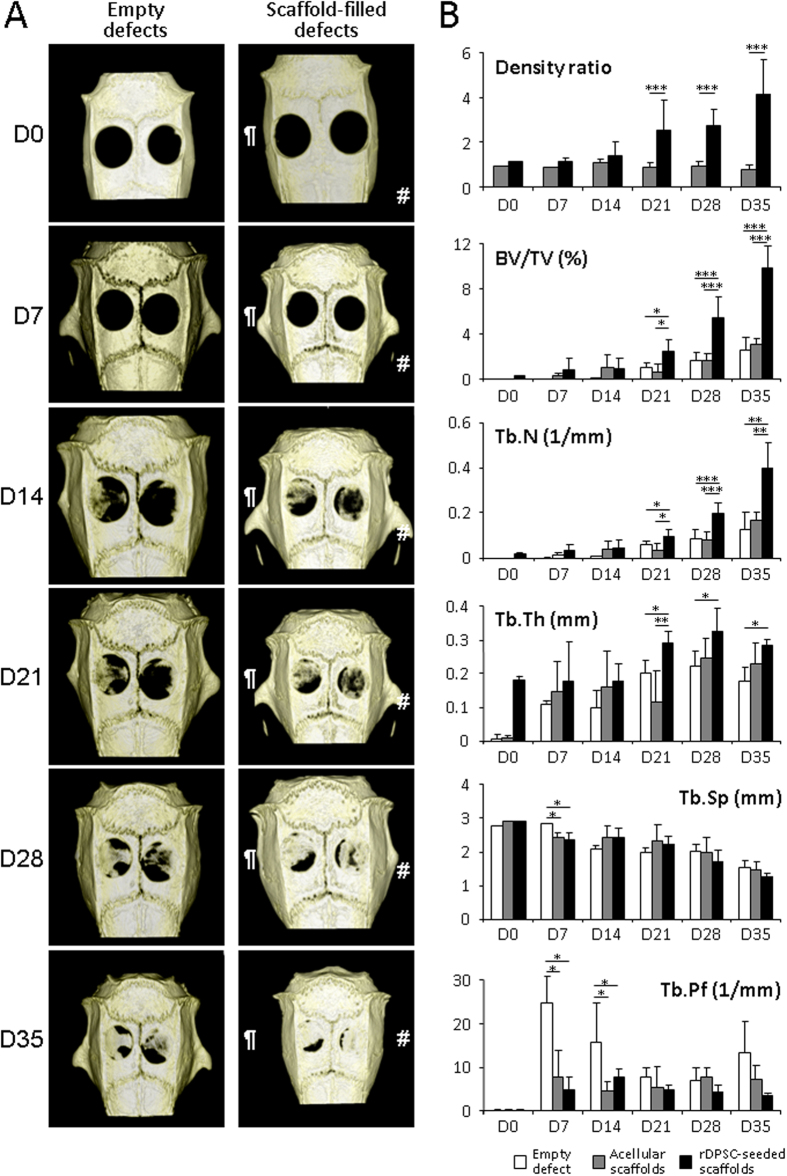
Micro-CT analysis (**A**) Rat skull 3D rendering at D0 to D35. Calvarial defects that were left empty did not heal spontaneously for the duration of the study. In contrast, bone healing was gradually achieved when the calvarial defects were filled with either rDPSC-seeded (#) or acellular dense collagen gel scaffolds (¶).(**B**) Bone density and micro-architectural parameters. Bone volumetric fractions were expressed as a percentage of bone volume on the total area of the defect. Bone parameters were quantified as a % of tissue volume (BV/TV) within the defected area, trabecular number (Tb.N), trabecular thickness (Tb.Th), trabecular separation (Tb.Sp) and trabecular pattern factor (Tb.Pf). *p < 0.05; **p < 0.01; ***p < 0.001.

To evaluate bone regeneration, the parietal bone was examined by micro-CT scan. Thirty-five days after surgery, control empty defects showed a thin non-mineralized healing connective tissue with no sign of bone regeneration. Parietal defects filled with either rDPSC-seeded or acellular dense gel scaffolds demonstrated a progressive amount of new bone formation starting from the edges as well as from the center of the defect. No inflammation was observed in the seeded scaffold implants. Micro-CT analysis of 3D micro-architecture parameters of the remodeled bone defect area are summarized in Fig. 2B. Compared to acellular scaffolds, denser and better-organized trabeculae were observed in rDPSC-seeded scaffolds whereas none of the defects showed complete closure at day 35 (Fig. 2A). Scaffolds seeded with rDSPCs demonstrated a significantly higher amount of calcified material within the remodeled defects when compared to acellular scaffolds from day 21 post surgery (density ratio of 2.5 ± 1.3 *vs*. 0.9 ± 0.2 at day 21, p < 0.05; and 4.2 ± 1.5 *vs*. 0.8 ± 0.2 at day 35, p < 0.01; respectively). Scaffolds with rDPSCs led to significantly higher mineralized tissue volume BV/TV and trabecular number Tb.N than acellular scaffolds or empty defects from day 21, without affecting the trabecular thickness Tb.Th, separation Tb.Sp and trabecular pattern factor Tb.Pf. In other words, the defected area acquired a higher mineralized healing tissue with higher trabecular structure when rDPSC-seeded scaffolds were used, suggesting a stimulatory effect of pulp stem cells on calvaria healing.

### Restored bone remodeling processes in scaffold-filled defects

**Figure 3:**
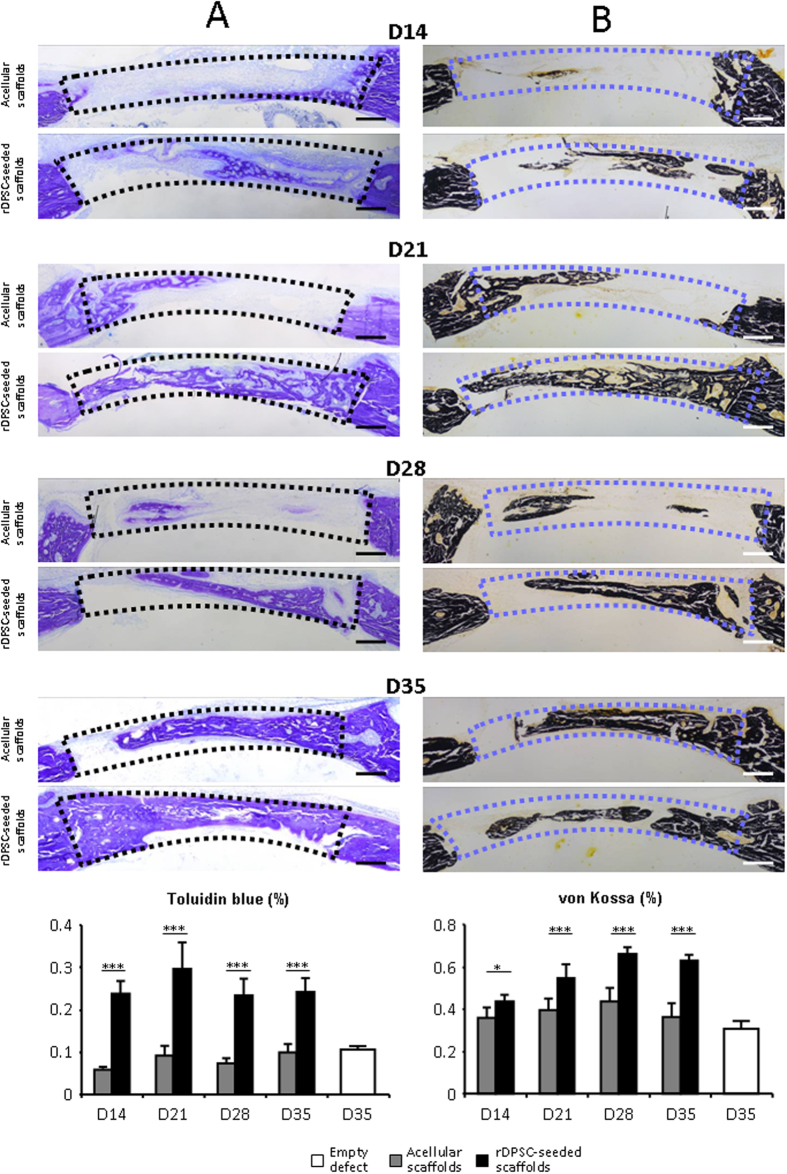
Histology and histomorphometry. Representative and resin embedded calcified bone stained with Toluidine Blue (**A**) and von Kossa (**B**) images are shown at days 14 to 35 post-operative for defects filled with rDPSC-seeded dense collagen gel scaffolds (n = 15) and acellular scaffolds (n = 15). Compared to acellular scafoolds, there was a significant increase in the percentage area (underlined by dotted lines) of Toluidine Blue and von Kossa positive staining in defects filled with rDPSC-seeded dense collagen gel scaffolds. No significant difference was observed at day 7 (data not shown). Scale bars represent 500 μm. *p < 0.05; ***p < 0.001.

To investigate the remodeled tissue within the bone defect area, fibrous connective tissue and mineralized deposits were stained with toluidine blue and von Kossa, respectively. New connective and bone tissue formation were calculated as a percentage of filling from the baseline defect. Histological evidence further supported the micro-CT findings, indicating that rDPSC-seeded scaffolds demonstrated a progressive presence of fibrous and mineralized connective tissue within the remodeled defect. The newly formed bone had a typical organized and mature bone morphology with noticeable marrow spaces and similar to native bone (Fig. 3). In contrast, histological analysis indicated a small amount of irregularly arranged bone tissue and less bone formation in acellular scaffolds. The percentage of new connective bone tissue visualized with toluidine blue area was significantly higher (p < 0.001) at days 14, 21, 28 and 35 post surgery when using rDPSC-seeded scaffolds, compared to both acellular scaffolds and empty defects. Similarly, the percentage of new mineralized tissue bone area evidenced with von Kossa was significantly higher at days 14 (p < 0.05) and 21, 28 and 35 post surgery (p < 0.001), when using rDPSC-seeded scaffolds, compared to both acellular scaffolds and empty defects.

**Figure 4:**
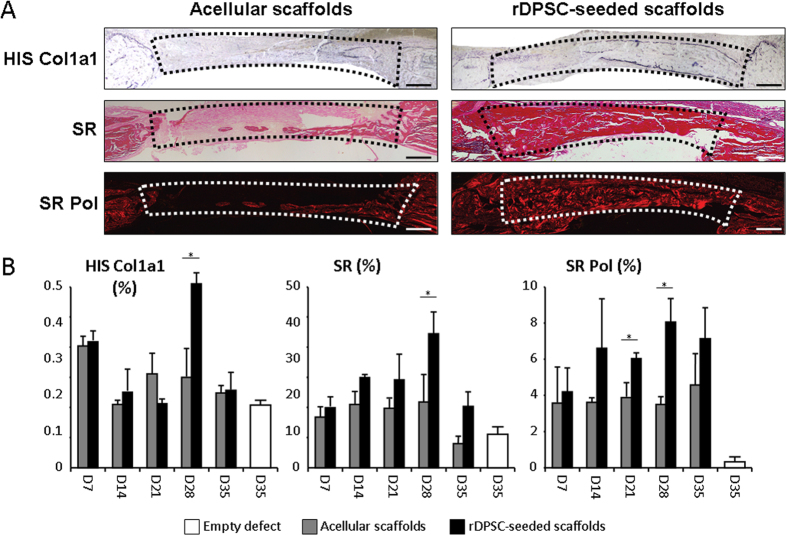
*In situ* hybridization and histology analysis of the collagen component during bone regeneration. *In situ* hybridization (HIS) studies using the Col1a1 probe reacting with recipient osteoblasts and osteocytes associated within the new bone formation showed strong collagen synthesis in defects filled with rDPSC-seeded dense collagen gel scaffolds, whereas only weak Col1a1-associated signals were observed in defects filled with acellular scaffolds. Sirius red (SR) stainings were performed to visualize the collagen protein neo-secretion when using polarized light (Pol). Representative images at day 28 post-operative (**A**) and quantification from days 14 to 35 (**B**) are shown for defects filled with rDPSC-seeded dense collagen gel scaffolds (n = 15) and acellular scaffolds (n = 15). Scale bars represent 500 μm. Dotted lines represent the analyzed areas. No significant difference was observed at day 7 (data not shown). *p < 0.05.

**Figure 5:**
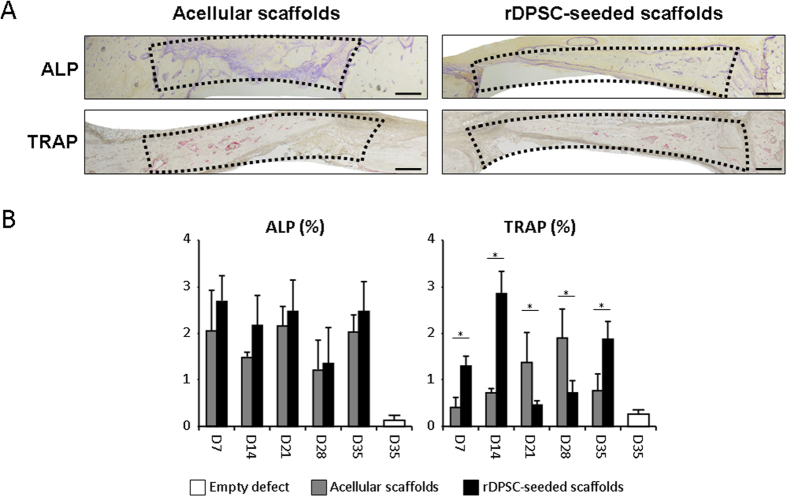
Immunohistochemical analysis of bone remodeling component during bone regeneration. Staining of osteoblastic-associated alkaline phosphatase (ALP) and osteoclastic-associated tartrate-resistant acid phosphatase (TRAP) activities revealed osteoblastic forming and osteoclastic resorbing processes within new mineralized bone tissue area, in particular when rDPSC-seeded scaffolds were implanted. Representative images at day 28 post-operative (**A**) and quantification from days 14 to 35 (**B**) are shown for defects filled with rDPSC-seeded dense collagen gel scaffolds (n = 15) and acellular scaffolds (n = 15). Scale bars represent 500 μm. Dotted lines represent the analyzed areas. No significant difference was observed at day 7 (data not shown). *p < 0.05.

*In situ* hybridization studies using the specific Col1a1 probe indicated strong collagen expression in rDPSC-seeded scaffolds, especially at the exterior and interior periosteal sides of the newly formed calvarium diploe, whereas only weak Col1a1-associated signals were observed in acellular scaffolds (Fig. 4). This increased Col1a1 gene expression was further supported with the comparison of Sirius red staining that marks the collagen fibers. This staining was much stronger in rDPSC-seeded scaffolds when compared with the acellular group, indicating that more collagen protein was secreted in the rDPSC group. In addition, the investigation of ALP and TRAP activities revealed a restored osteoblastic forming and osteoclastic resorbing processes within new mineralized tissue bone area, especially when rDPSC-seeded scaffolds were implanted (Fig. 5).

## Discussion

Craniofacial area is particularly vulnerable to defects or bone loss because of trauma, pathologies or hereditary malformation, for which bone reconstruction is particularly difficult. Here, we show that dense collagen scaffolds combined with mesenchymal stem cells derived from the dental pulp drastically enhanced bone regeneration in a calvarial defect model, suggesting that this association represents an effective therapeutic tool to regenerate craniofacial bones.

In this study, a dense collagen gel scaffold was used as a carrier for CD31^−^ CD45^−^ CD73^+^CD90^+^ rDPSCs to assess their bone regeneration effects on the very thin rat skull. This naturally-derived biomimetic biomaterial, that resembles the organic or “osteoid” phase of native bone, was simply generated through rapid removal of fluid from highly-hydrated collagen gels using plastic compression^13^, where cell seeding is part of the scaffolds processing. This natural scaffold has been reported to exhibit osteoinductive and osteoconductive abilities^14^,^15^,^34^,^41^,^42^ that are critical for osteointegration into the bone, especially for craniomaxillofacial applications. Through the increased collagen fibrillar density, a native matrix scaffold that mimics the microstructure (and stiffness) of the osteoid is produced, which provides both biological and mechanical cues to accelerate osteogenic differentiation of three-dimensionally seeded cells. Furthermore, the clinical use of collagen scaffolds is successful and widely accepted with excellent biodegradation, and biocompatibility^43^,^44^,^45^. In particular, previous studies have shown that collagen-based scaffolds are substitutable materials^46^, which in combination with MSCs can promote mineralization and heal a large bone unicortical defect^16^. In this study, authors used osteogenic pre-conditioned mesodermal bone marrow derived MSC, seeded into a compressed type I collagen scaffold, to heal a mesodermal appendicular weight bearing bone (femur) after an unicortical defect in the mouse. Furthermore, VEGF was injected into the femoral canal at the level of the defect to enhance angiogenesis^16^. Here, both the mesenchymal cell embryologic origin and the damaged bone specificity differed from this previous study. Indeed, we evaluated for the first time the osteogenic effects of the association of a dense collagen gel scaffolds seeded with non-pre-conditioned neural crests derived DPSCs on bone regeneration in a rat critical-size calvarial defect model, a non-weight-bearing bone formed by intramembranous ossification. In addition, no VEGF was used to enhance angiogenesis.

Critical-sized bone defect are currently the reference models in exploring the efficiency of osteoconductive substitutes on healing and regeneration^36^. The bilateral rat calvarial defect model used in this study is highly reproducible. Two critical-sized defects were created on either side of the parietal region and filled with either rDPSC-seeded, or acellular dense collagen gel scaffold, whereby each rat was its own control. At day 35 post surgery, rDPSC-seeded dense collagen gel scaffold displayed osteogenic effects enhancing bone healing, which was in contrast to the control group that did not show bone regeneration, confirming that the selected defect model was critical over the duration of the study.

Contrary to a previous report^31^, graft rejection was not observed in the present study. Stem cells from dental sources are interesting with the ease of accessibility, and high proliferation/differentiation capacities^47^. Indeed, osteogenic differentiation of DPSCs seeded in dense collagen gel scaffolds was another important factor in the potential success of bone regeneration. Since their first isolation in 2000^24^, DPSCs deriving from neural crests^22^ have been reported to have a higher proliferation rate than bone marrow stem cells^5^ and adipose-derived stem cells^48^,^49^. Similar to mesenchymal stromal cells, DPSCs are reported to be positive for CD73 and CD90 (two mesenchymal stem cell markers), and negative for CD31 (a marker of endothelial lineage) and CD45 (a marker of hematopoietic lineage)^21^,^22^. Furthermore, autologous DPSCs have been shown to promote bone regeneration when used in association with a collagen sponge^6^. Since wisdom teeth extraction is one of the most common procedures in oral surgery, the harvested DPSCs can be *per se* considered as a powerful source of stem cells useful for autologous tissue engineering^50^ with a positive impact on bone regeneration^32^,^51^,^52^. Pathologic features of the craniomaxillofacial skeleton, also mainly a neural crest-derived tissue, could possibly be corrected with stem cell therapies. Owing to their similar embryonic origins, the potential of DPSCs as an autologous stem cell source for treating skull defects was investigated.

The samples in this study were investigated both by histology and by micro-computed tomography. Micro-CT analysis has become a major technique of investigation in the assessment of bone regeneration/healing in bone defect models, with several advantages such as rapid calculation and multiple analysis opportunities^20^,^53^. Histomorphometric examinations are reliable and widely used in the assessment of bone tissue regeneration/healing. In the current study, the dense collagen-based scaffold supported the long-term metabolic activity of DPSCs to promote the recruitment and differentiation of osteoblasts that deposited a calcium-phosphate rich mineral in the extracellular matrix, associated with the expression of type I collagen. The presence of a fibrous and mineralized scaffold, stained with toluidine blue and von Kossa, localized in the centre of the defect indicated that osteoinduction was, at least in part, initiated or promoted by cells trapped in the scaffold. This was also further supported by the strong osteoblast-associated ALP staining within and at the periphery of the healing defect. Together with the presence of TRAP-positive osteoclasts in and around the scaffold, this suggests an early active and restored matrix turnover within the initial defect, especially when rDPSC-seeded dense scaffolds were used. The osteoconductive properties of the mineralized scaffolds, aided by DPSCs, would thus enable migration and attachment of endogenous bone forming progenitor cells. The presence of both osteoblasts and osteoclasts in central and peripheral areas further suggests the extracellular matrix within the bone defect was undergoing active turnover. Additional studies aimed at determining the fate of transplanted DPSCs and their relationship with endogenous cells during defect repair will require the use of a tracking system or other labeled cells in the scaffold. A detailed time course, with long-term bone healing effects (i.e. >5 weeks) will be necessary to reveal the molecular mechanisms underlying the increase in bone formation observed in the current study at one month.

## Conclusions

This study has demonstrated the potential of neural crest derived DPSC-loaded-dense collagen gel scaffolds to benefit the craniofacial bone healing process. The association of DPSCs with appropriate physiological osteogenic scaffold (dense collagen gel) is a promising approach when using tissue-engineering techniques for clinically relevant large craniofacial bone defects and opens new avenues for further research aiming to improve cell cultivation and scaffold design.

## Additional Information

**How to cite this article**: Chamieh, F. *et al*. Accelerated craniofacial bone regeneration through dense collagen gel scaffolds seeded with dental pulp stem cells. *Sci. Rep.* **6**, 38814; doi: 10.1038/srep38814 (2016).

**Publisher's note:** Springer Nature remains neutral with regard to jurisdictional claims in published maps and institutional affiliations.
